# Using machine learning to generate high-resolution wet area maps for planning forest management: A study in a boreal forest landscape

**DOI:** 10.1007/s13280-019-01196-9

**Published:** 2019-05-09

**Authors:** William Lidberg, Mats Nilsson, Anneli Ågren

**Affiliations:** 1 Department of Forest Ecology and Management, Swedish University of Agricultural Sciences, Umeå, Sweden; 2 Department of Forest Resource Management, Swedish University of Agricultural Sciences, Umeå, Sweden

**Keywords:** Digital elevation model, LiDAR, Machine learning, Random Forest, Soil classification, Wet area mapping

## Abstract

Comparisons between field data and available maps show that 64% of wet areas in the boreal landscape are missing on current maps. Primarily forested wetlands and wet soils near streams and lakes are missing, making them difficult to manage. One solution is to model missing wet areas from high-resolution digital elevation models, using indices such as topographical wetness index and depth to water. However, when working across large areas with gradients in topography, soils and climate, it is not possible to find one method or one threshold that works everywhere. By using soil moisture data from the National Forest Inventory of Sweden as a training dataset, we show that it is possible to combine information from several indices and thresholds, using machine learners, thereby improving the mapping of wet soils (kappa = 0.65). The new maps can be used to better plan roads and generate riparian buffer zones near surface waters.

## Introduction

Open peatlands are a recognizable feature in the boreal landscape that are commonly mapped from aerial photographs. However, wet soils also occur on tree covered peatlands (Creed et al. 2003) and in the riparian zones of forest streams and surrounding lakes (Gregory et al. 1991). Wet soils have lower bearing capacity than dry soils (Cambi et al. 2015) and are more susceptible to soil disturbance from land-use management with heavy machinery (Mohtashami et al. 2017). Off-road driving with heavy machines can cause wet soils to deform and displace resulting in deeper tracks and larger soil disturbance than on dry soils where shallower tracks are caused by compaction. Forestry conducted close to streams and lakes has been shown to increase the export of mercury (Eklöf et al. 2016) and nutrients (Kreutzweiser et al. 2008) to downstream environments (Kuglerová et al. 2014). Soil damage in riparian zones can also lead to erosion from ruts and subsequent sediment deposition burying important spawning habitats (Kreutzweiser and Capell 2001). Forested buffer zones and machine free areas near streams and lakes are commonly used to protect surface water during forestry activities but implementing these protective measures in practice can be complicated due to poor planning tools. For example, Ågren et al. (2015) compared manually mapped streams to current maps and concluded that 60% of the perennial stream network and 80% of all streams are missing from current maps in Sweden. This makes it difficult for managers to plan off-road driving and protective measures, particularly buffer zones around streams (Laudon et al. 2016; Kuglerová et al. 2017). Kuglerová et al. (2017) argued that buffer zones around streams should take small-scale hydrologically active areas into account but without accurate maps of these variations it cannot be implemented in practice.

Topographical modelling of wet area indices has been suggested as a solution to this problem (Murphy et al. 2008; Ågren et al. 2014) and high-resolution digital elevation models (DEM) derived from Light Detection And Ranging (LiDAR) are becoming accessible in many countries, making this a popular approach (van Leeuwen and Nieuwenhuis 2010; Guo et al. 2017). Topographic wetness index (TWI) (Beven and Kirkby 1979) is often used to map wet areas but is sensitive to DEM resolution (Ågren et al. 2014) as well as which algorithms are used to calculate TWI (Sørensen et al. 2006). Creed and Beall (2009) later built on TWI with variable source area (VSA) to map cryptic wetlands and predict nitrogen transport to streams in Canada. Hjerdt et al. (2004) suggested downslope distance or downslope gradient index but this method requires catchment-specific thresholds to define wet areas. Wet area indices based on stream networks, such as elevation above stream (EAS) (Rennó et al. 2008) and cartographic depth to water (DTW) (Murphy et al. 2008), have already proven to be useful and DTW maps are used today in, for example, Sweden and Canada to plan forestry operations. However, since they are based on stream networks, it is necessary to define a stream initiation threshold, something that has proven to be difficult due to temporal dynamics (Ågren et al. 2015) and spatial distribution of soils types (Ågren et al. 2014). An early attempt to include soil transmissivity in TWI was done by Beven (1986) and more recent attempts include both soil and climate (Güntner et al. 2004). Most of these topographical methods rely on the user to define appropriate threshold values in order to define wet areas. Ågren et al. (2014) demonstrated that the optimal flow initiation threshold used to extract depth to water maps (DTW) varied greatly even on a local scale. Soil textures, topography and climatic differences make any application difficult on a large scale. To handle these limitations, new methods are necessary. Such new methods include the use of machine learning (ML) in digital soil mapping (Maxwell et al. 2018). ML is a data mining technique that finds patterns in datasets and uses these patterns to predict new data. Several ML algorithms are available (Hastie et al. 2009) but the optimal method depends on the nature of the problem and it is usually recommended to explore several algorithms (Maxwell et al. 2018).

The aim of this study is to evaluate how ML and data from national inventories from productive and non-productive forest land can be used with wet area indices and existing map data to generate more accurate maps of wet soils on a high resolution that can be used to plan forestry operations.

## Materials and methods

### Study site

Sweden is situated in Northern Europe between latitude 55° and 70°N and longitude 11° and 25°E, which means that most of the country is within the boreal zone. Sweden is to 75% covered by glacial till, while peat is the second most dominant soil type and covers 13% of Sweden (Fransson 2018). According to the Swedish Land Cover database (based on satellite imagery) (Ansén 2004), the land cover in Sweden is as follows: forest 63.0%, lakes 8.9%, open mire 8.7%, heathlands 7.7%, arable land 6.1%, forested mire 2.8%, urban areas 2.3% and other 0.6%. However, the NFI estimates that 67% of Sweden is forest land (Fransson 2018).

### Field data

**Fig. 1:**
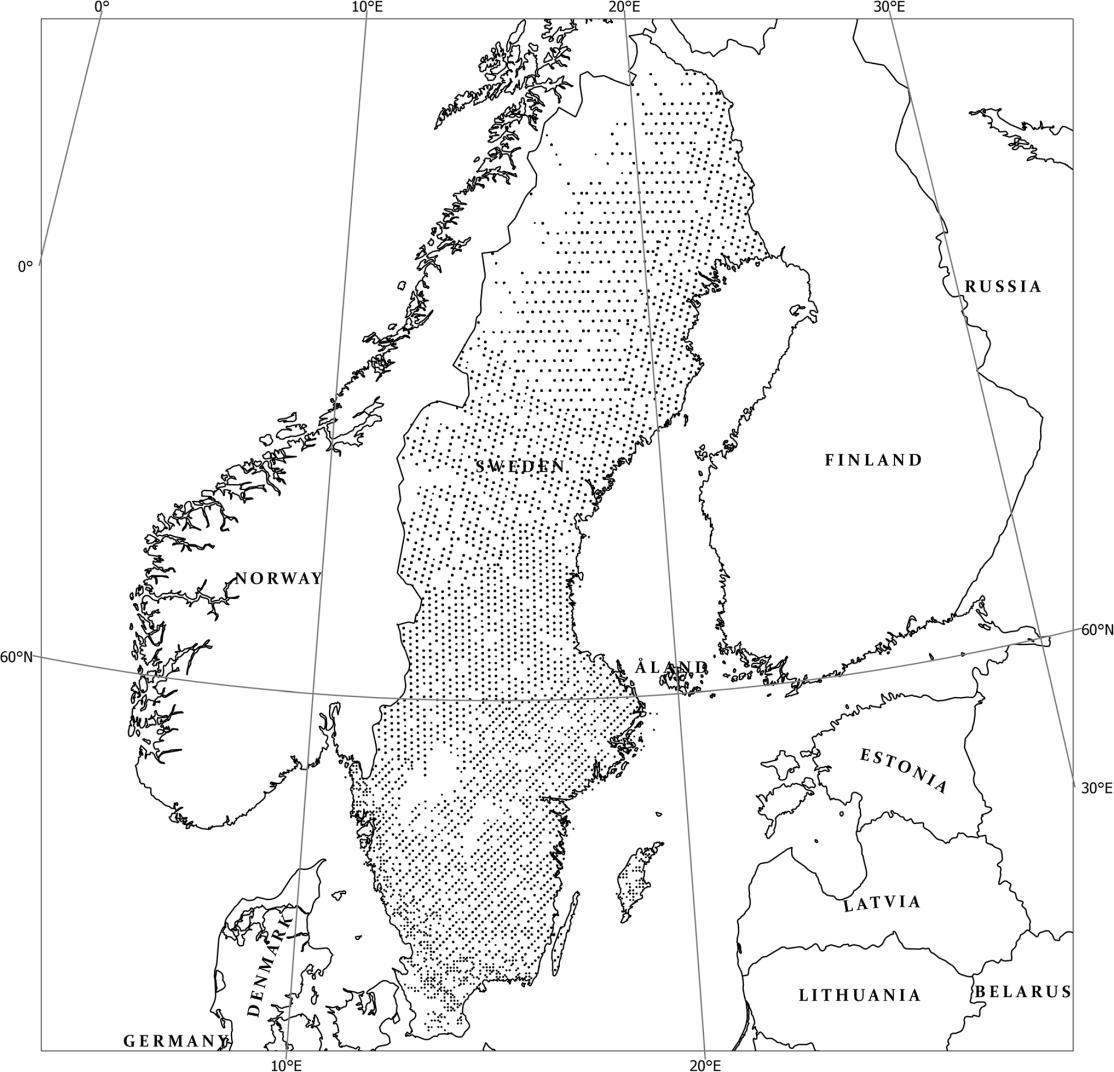
The 19 645 NFI field plots are marked with black points. The density of field plots are higher in southern Sweden than northern Sweden and the white regions in northwestern Sweden were not yet scanned with LiDAR at the time of this study

The Swedish National Forest Inventory (NFI) started in 1923 and contains both permanent plots with a radius of 10 m and temporal plots with a radius of 7 m. Only permanent plots inventoried between 2012 and 2016 were used for this study due to better accuracy in GPS positioning than temporal plots. The accuracy of the included plots was within 5–10 m. The NFI includes a random sampling of both productive forest land (defined as areas with a potential yield capacity of > 1 m^3^ mean annual increment per ha) and low-productive forestland (potential yield capacity of < 1 m^3^ mean annual increment per ha), for example, peatlands, pastures, thin soils, rock outcrops and areas close to and above the tree line. However, crop fields, urban areas, roads, railroads and power lines are excluded from the random sampling. This means that the registrations of soil moisture give a good representation of the distribution of soil moisture in the landscape outside of urban and arable areas. Further, only sites covered by the Swedish National DEM could be included in this study resulting in 19 645 plots (Fig. 1). These plots where used as training data for the machine learning classification described in the classification section below.

The NFI registers average soil moisture condition in each plot based on vegetation patterns and the position in the landscape in five classes: dry (6%), mesic (54%), mesic–moist (27%), moist (10%) and wet (3%) (out of the sample plots on productive and non-productive forest land). Estimating soil moisture from vegetation is a way to ignore temporal variations and instead determine the general wetness regime. A short description on each soil class follows:

- Wet soils are normally soils located on open peatlands that are classified as bogs or fens, where trees can occasionally occur but not in dense stands. The groundwater table is close to the soil surface and permanent ponds are common; soils are histosols or gleysols. The thickness of the organic layer is often > 30 cm. One cannot walk dry footed on wet soils and it is often not possible to cross wet soils with heavy machinery unless soils are frozen during winter.
- Moist soils are areas with a shallow groundwater level (< 1 m). Pools of standing water are visible in local pits. It is possible to cross these areas dry footed in low shoes if you utilize higher lying areas and tussocks; however, a pool of water should form around the shoe in lower laying areas, even after dry spells. Soils are histosols or gleysols, and they can also be categorized as regosols which is a taxonomic rest group. Vegetation is dominated by wetland mosses (e.g. *Sphagnum* sp., *Polytrichum commune, Polytrichastrum formosum*, *Polytrichastrum longisetum*) and *Sphagnum* sp. dominates local depressions. Trees show a coarse root system above ground and tussocks are common indicating an adaption to high groundwater levels in these areas. The thickness of the organic layer is not used to define moist areas but it is often > 30 cm.
- Mesic–moist soils are areas where the groundwater table is on average less than 1 m from the soil surface, normally flat areas on lower laying grounds or on lower parts of hillslopes. These soils wet up on a seasonal basis following snowmelt or rain. If you can cross these areas dry footed or not depends on the season. Wetland mosses (e.g. *Sphagnum* sp., *Polytrichum commune*, *Polytrichastrum formosum*, *Polytrichastrum longisetum*) are common and trees show a coarse root system above ground indicating that high groundwater levels are common in these areas. Soils are humo-ferric to humus-podzols. The organic soils are thicker than on mesic soils and while podzols are common the O-horizon is still often peaty (peaty moor).
- Mesic soils consist of ferric podzols with a thin humus layer covered by mainly dry land mosses (e.g. *Pleurozium schreberi*, *Hylocomium splendens*, *Dicranum scoparium*). The groundwater table is on average 1–2 m below the soil surface. Here you can walk dry footed even directly after rain or shortly after snowmelt. The organic layers are normally 4–10 cm.
- Dry soils have the groundwater table at least 2 m below the surface. They tend to be coarse textured and can be found on hills, eskers, ridges and marked crowns. Soils are leptosols, arenosols, regosols or podzols (the podzols have thin organic and bleached soil horizons).

Here we focus on a forest management perspective, where the main aim is to generate a map for forest soil trafficking. Wet soils are too wet to drive on unless frozen or using technical aids. While it is possible to cross moist soils and mesic–moist soils with heavy machinery, it is best to avoid them since they have a relatively low bearing capacity. The high wetness and high organic content of moist soils and mesic–moist soils makes them deform and displace easily, causing more soil disturbance and deeper rut formation compared to the dryer more minerogenic dry and mesic soils where the tracks are shallower and normally only formed due to compaction of soils (Williamson and Neilsen 2000). Therefore, we divided the NFI dataset into two categories, “wet” and “dry”. Dry and mesic plots were classified in the “dry” category (60% of the NFI plots) while mesic–moist, moist and wet plots were classified in the “wet” category (40% of the NFI plots). This means that the “wet” category contains more mesic–moist plots than actual wet plots. Mesic–moist soils is not normally associated with open peatlands or wetlands but the definition of soils < 1 m depth to the groundwater table as unsuitable for trafficking also agrees with previous wet area mapping to define wet soils (Murphy et al. 2008; Ågren et al. 2014). We argue that “wet” soils are more sensitive to runt formation and it is better to traffic “dry” soils. To avoid confusion we write wet when we mean a more general description of wet conditions, and “wet” when we refer to new binary “wet”/“dry” grouping described above; this agrees with the terminology used in previous studies on wet area mapping (Murphy et al. 2008; Ågren et al. 2014); however, “wet” soils are not necessarily wet, per se.

### Variables derived from the digital elevation model

To locate “wet” soils, several terrain indices were calculated that predict the location of “wet” soils based on the assumption that topography controls the groundwater flow. This study used the Swedish National DEM generated by the Swedish Mapping, Cadastral and Land Registration Authority using LiDAR data. This DEM has a cell resolution of 2 m × 2 m and was generated from a point cloud with a point density of 0.5–1 points m^−2^ with a horizontal and vertical error of 0.1 m and 0.3 m, respectively. The DEM was split into 2818 sub-catchments where each catchment had 2 km overlap with surrounding catchments to avoid edge effects when extracting streams. These sub-catchments were processed separately for topography (Local topography section), elevation above stream (Elevation above stream section) and depth to water (Depth to water section) and the outputs were mosaicked back together before the values were extracted to the field plots.

#### Local topography

Local topography is recognized as an important factor for controlling soil moisture (Moeslund et al. 2013) and one way to extract values of local topography is to use the standard deviation of elevation from a DEM. Here a moving window with 5 × 5, 10 × 10, 20 × 20, 40 × 40 and 80 × 80 grid cells was used to calculate the standard deviation of elevation at each field plot. High values indicate steep terrain, while low values indicate flat terrain.

#### Topographical modelling to extract wet soils

The DEM was preprocessed using a three-step breaching approach developed in Lidberg et al. (2017) in order to become hydrologically correct before it was used for hydrological modelling. Lidberg's approach was developed to be a reliable approach to correct the 2 m × 2 m Swedish DEM.

A flow pointer grid and a flow accumulation grid were extracted from the hydrologically correct DEM using Deterministic-8 (D8) (O’Callaghan and Mark 1984). D8 was chosen since it is computationally effective and the difference to more complex flow routing algorithm has been shown to be limited on high-resolution DEMs (Leach et al. 2017). Streams were then extracted from the flow accumulation grid using stream initiation thresholds of 0.5 ha, 1 ha, 2 ha, 5 ha, 10 ha, 15 ha and 30 ha. Lake and river polygons from the property map were converted to raster and merged with the previously extracted raster streams in order to create source layers with cells that represent surface water.

#### Elevation above stream

Elevation above stream (EAS) is calculated using the source layer containing surface water described above, the same D8 pointer grid as used to extract streams, and the original DEM. The elevation above stream is calculated as the difference in elevation between a grid cell in the landscape and its nearest source cell that represents surface water, measured along the downslope flow path determined by the D8 pointer grid (Rennó et al. 2008). This was done for each of the source layers with the same stream initiation thresholds as mentioned above.

#### Depth to water

Depth to water (DTW) is similar to previously described elevation above stream since both calculate an elevation difference from a source grid to surrounding landscape. The difference is that depth to water calculates the elevation along the least-cost-path instead of the downslope flow path determined by the D8 grid. The cost is the slope of the DEM calculated by the Eq. (1) as described by Murphy et al. (2008).

1 $$ {\text{DTW}}\;(m) = \left[ {\sum \frac{{{\text{d}}z_{i} }}{{{\text{d}}x_{i} }}a} \right]xc, $$

where d*z*/d*x* is the slope of a cell along the least-elevation path, _*i*_ is a cell along the path, *a* equals 1 when the path crosses the cell parallel to cell boundaries and $\sqrt 2$ when it crosses diagonally; *xc* represents the grid cell size (m).

#### Topographic wetness index

Topographic wetness index (TWI) describes how likely an area is to be wet based on its specific catchment area and local slope as described in Eq. (2), where *As* is the specific catchment area and *slope* is the slope of the grid cells in degrees (Beven and Kirkby 1979).

2 $$ {\text{TWI}} = { \ln }\left( {\frac{As}{{\tan ( {\text{slope}})}}} \right). $$

In this study, it was calculated using the D-infinity flow routing algorithm (Tarboton 1997) which is better than D8 on coarser grids, and the wetness tool in Whitebox GAT 3.4. Since TWI is scale dependent, we resampled the 2 m DEM to a 24 m DEM and a 48 m DEM as these have been found to be suitable resolutions for TWI calculations in the forested Krycklan catchment in northern Sweden (Ågren et al. 2014).

### Other factors affecting the hydrological modelling

**Fig. 2:**
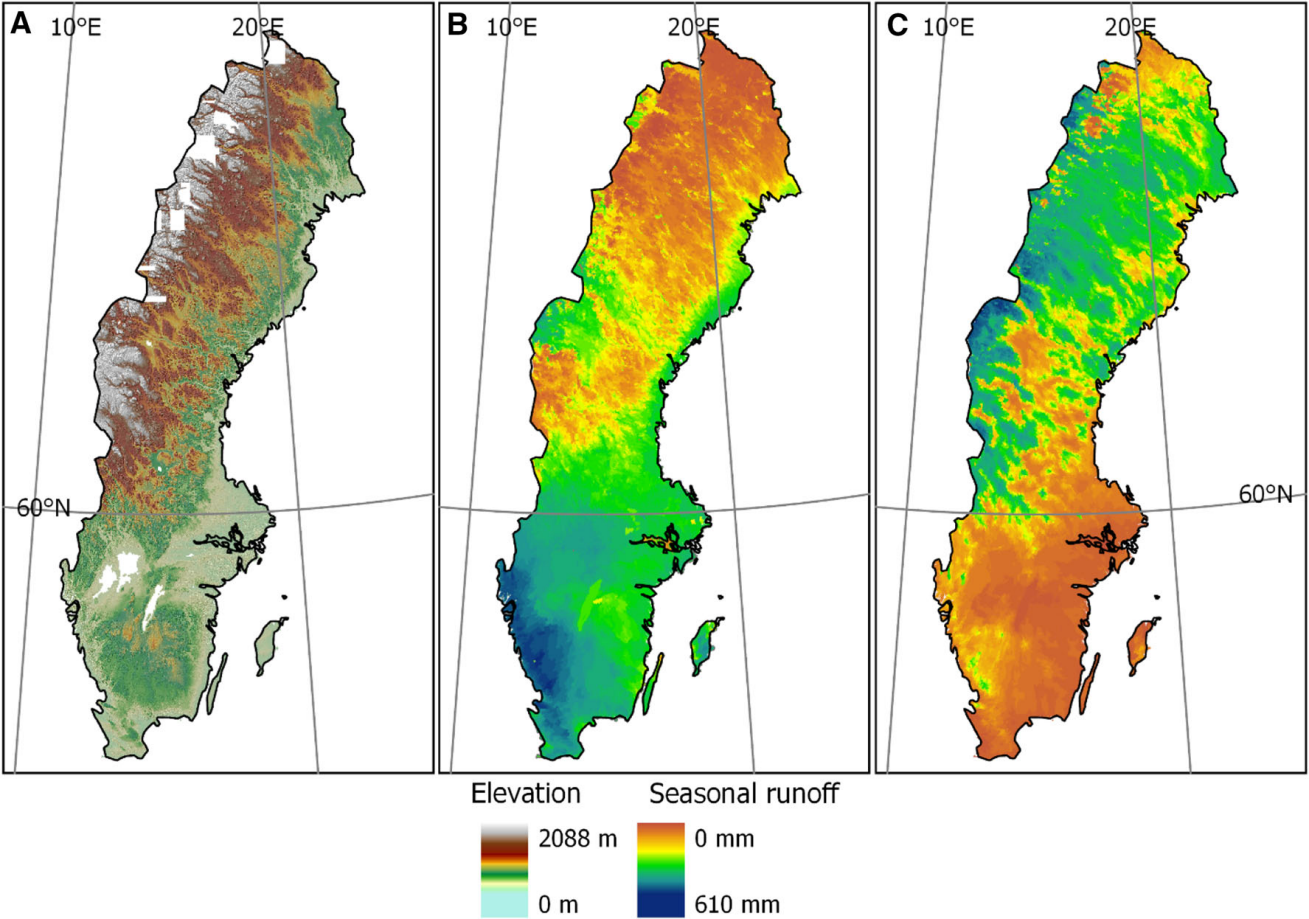
An example of the variability of the landscape and climate in Sweden that could affect the hydrological modelling (“Other factors affecting the hydrological modelling”). Here exemplified by (A) the Swedish national DEM, (B) average winter runoff from the last 30 years and (C) average spring runoff from the last 30 years

The quaternary deposit is an important factor for soil moisture since it determines the permeability and drainage capacity of soils. Quaternary deposits were extracted from maps created by the Swedish Geological Survey. There are several maps of quaternary deposits in Sweden and the scale and coverage of these maps ranges from 1:25 000 (1.7%), 1:50 000 (2.7%) 1:100 000 (47%), 1:200 000 (1.4%), 1:250 000 (21.2%), 1:750 000 (33.6%) and 1:1 000 000 (100%) (GET 2018). Some of these maps have significant overlap but the highest resolution map was always chosen in the overlapping areas. The quaternary deposits were merged by hydrological function into five main categories: till soils, peat soils, course sediments, fine sediments and rock outcrops. Additionally open wetlands are more accurately mapped on the 1:12 500 scale property map so these were used in addition to the peat layer from the quaternary deposits map. There is considerable variability in runoff between different regions in Sweden and across seasons (Fig. 2). A high runoff should reflect higher groundwater levels which in turn could affect the distribution of wet soils. S-HYPE (Arheimer et al. 2011) was used to model seasonal and annual runoff in 33 605 sub-catchments between 1982 and 2015. These variables will be referred to as “Spring”, “Summer”, “Autumn”, “Winter” and “Average”.

### Machine learning classification of wet areas

There are many different ML algorithms available (Hastie et al. 2009) and their use for soil classification has already been evaluated (Maxwell et al. 2018). Four commonly used ML algorithms were tested to generate predictions of “wet” and “dry” soils: artificial neural network (Ripley 1996), random forest (Breiman 2001), support vector machine (Chang and Lin 2011) and naïve Bayes classification (Bhargavi and Jyothi 2009). The R package “Caret” (Kuhn et al. 2012) was used to evaluate all machine learners. Multicollinearity among variables was tested and variables with a correlation over 0.9 were excluded prior to analysis. The NFI dataset was split, randomly, into 75% training data and 25% test data and all ML algorithms were parameterized and tuned using a grid-search approach in combination with 10-fold cross-validation to find the best-fitting model. The tuned models were applied on the test dataset and evaluated using Cohen’s kappa index of agreement.

Visual examination of maps has proved to be essential for assessing spatial ML predictions (Maxwell et al. 2018). Therefore, as a compliment to the statistical results that were based on the NFI test plots, we also applied the trained models to classify soil moisture in the Krycklan catchment (Laudon et al. 2013). This catchment was chosen because the authors are familiar with the area and have conducted research there for over a decade (Hasselquist et al. 2017). Wet areas and riparian zones have been mapped (Ågren et al. 2014), groundwater hot spots have been investigated (Leach et al. 2017), and culverts (Lidberg et al. 2017) have been mapped as well as temporal dynamics in the stream network (Ågren et al. 2015). The maps were used for visual inspection and compared to first-hand knowledge of the area.

### Comparison with currently available maps

**Fig. 3:**
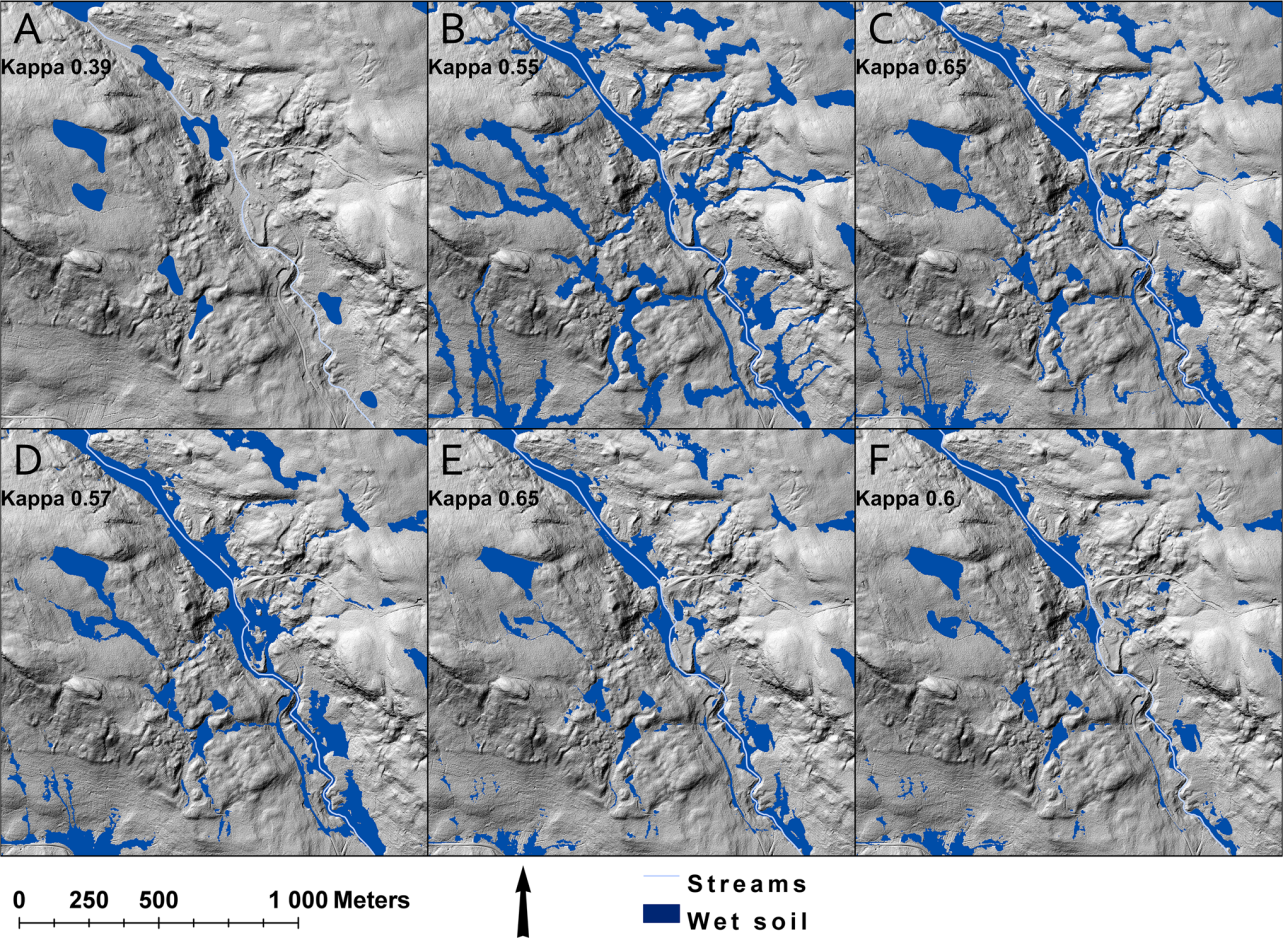
Wet areas are superimposed on a hillshade of a DEM in the Krycklan catchment. (A) The wetlands from the property map, this map misses many of the wet areas. (B) Swedish Forest Agency DTW map, this map has tendency to be too wet. Panel C–F shows the “wet” class using different machine learners: (C) random forest, (D) naïve Bayes, (E) artificial neural network, (F) support vector machine. Even the worst ML map (naïve Bayes) performed better than the SFA DTW map, but random forest and artificial neural networks generated the best results. The kappa values stated in the panels represents the maps performance for the entire forest landscape, even though the panels are zoomed into a very small subsection of the Krycklan catchment

To be able to compare the performance of the ML wet area maps with today’s wet area maps, their performance was also calculated (Table 2). We present data on the wet areas on the highest resolution map covering all of Sweden, the Swedish Property map (1:12 500) from Swedish Mapping, Cadastral and Land Registration Authority. In 2015, the Swedish Forest Agency (SFA) introduced a DTW map that is accessible online to private Swedish forest owners. The DTW map used by the SFA was calculated by setting two thresholds: the stream network initiation threshold which was set to 1 ha and the wet soil threshold defined as the depth to the modelled groundwater surface which was set to ≤ 1 m. These maps are presented in Table 2 and Fig. 3 as reference.

## Results

**Fig. 4:**
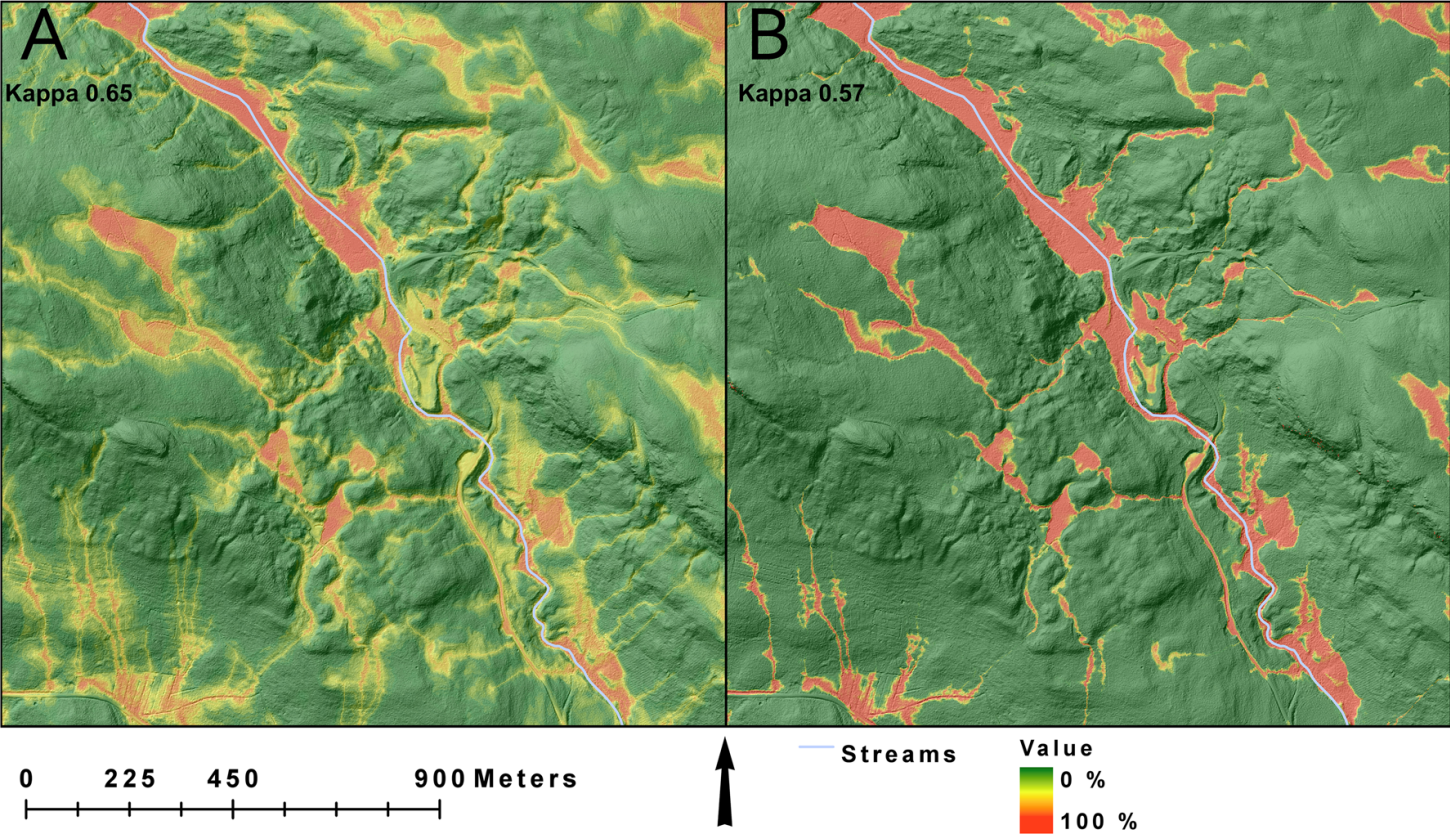
Example of probability (%) of predicted “wet” areas for one of the most accurate learner and the least accurate learner: (a) random forest and (b) Naïve Bayes. Areas with high probability of being classified as “wet” are coloured in red, while areas with low probability of being classified as “wet” are green. The yellow areas in between are where the models are uncertain whether they should be classified as “wet” or “dry”

The wet area map from the property map (Fig. 3a) only correctly classified 36% of all “wet” field plots (Table 2). In total, it classified 74% of “dry”/”wet” areas correctly (Table 2). The introduction of the SFA DTW map (Fig. 3b) meant that the accuracy of the wet area maps improved and correctly classified 73% of all “wet” field plots but it also classified 17% of all “dry” field plots as “wet” (Table 2), indicating that the SFA DTW map is too wet. The ML maps (3C-F) performed even better, where random forest (Fig. 3c) and artificial neural networks (Fig. 3e) produced the best maps and classified 84% of the “dry”/“wet” soils correctly (Table 2). Some ML models also have the ability to map probability of their classifications. Figure 4 shows the probability (%) of an area being classified as “wet”.

**Fig. 5:**
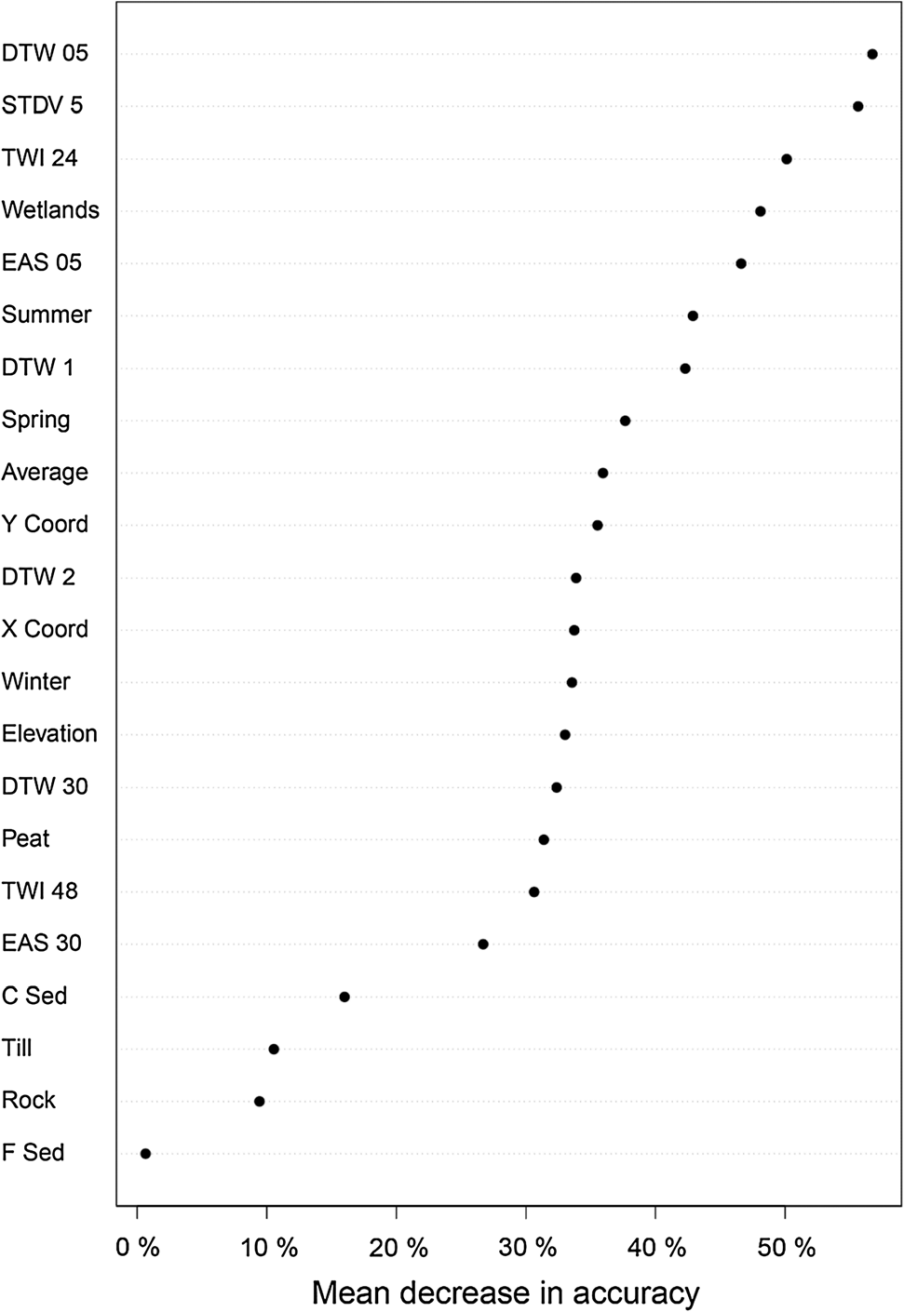
Important variables for each random forest model. The importance is measured as mean decrease in accuracy of the RF model if the variable is excluded. Higher values indicate important variables. “DTW X” and “EAS X” refer to depth to water and elevation above stream where X is the stream initiation threshold they are based on. “TWI 24” is topographical wetness index from a 24 m DEM, while “TWI 48” is TWI from a 48 m DEM. “STDV” stands for standard deviation of elevation and the number specifies how many cells in the DEM were used in the moving window. “Spring”, “Summer”, “Autumn”, “Winter” are average seasonal runoff, while “Average” is average annual runoff. “Wetlands” refer to the wetland layer from the property map, while the quaternary deposits are labelled “Till”, “Rock”, “Peat”, “C_Sed” (Course sediment), “F Sed” (Fine sediment). Finally “X_Coord”, “Y_Coord” and “Elevation” are the coordinates and elevation of the field plots. Variables that were described in the method section but not listed in Fig. 5 were removed from the dataset due to multicollinearity

All variables do not contribute equally to the predictive power of the model. The importance of a variable can be estimated based on how much the accuracy decreases when the variable is excluded from the learning process. Excluding an important variable will reduce the accuracy of the model. Excluding a less important variable will have a smaller impact on the accuracy since that variable contributed less to the predictive power. For example in the case of random forest, the three most important variables were standard deviation from elevation using a 5 cell moving window, a DTW map with 0.5 ha stream initiation threshold, and topographic wetness index from a 24 m DEM, while the least important variables were the quaternary deposits (Fig. 5).

## Discussion

Several studies, worldwide, have pointed out that current maps over wet areas (Murphy et al. 2008) and stream networks (Benstead and Leigh 2012) are lacking in accuracy and suggest modelling from a DEM as a way of generating better maps. Here we found that only 36% of all “wet” field plots were mapped as wetlands on the property map (Table 2) and since open wetlands and mires are easy to distinguish from aerial photos it is likely that the remaining 64% of the “wet” plots are located on tree covered wetlands and in riparian zones. DTW maps have been introduced in Sweden and Canada as forest management planning tool (Ågren et al. 2014, 2015; Murphy et al. 2008). Indeed, the SFA DTW map performed better, but had a tendency towards being too wet since it had the lowest accuracy for “dry” field plots (Table 2). The major improvement with the SFA DTW map was that this map also included wet areas near streams, the riparian soils (Fig. 3b). However, there is regional and local variability in stream networks and extent of riparian soils depending on climate, soil permeability and terrain topography (Fig. 2) (Ågren et al. 2014). This complex landscape variability can be captured by utilizing machine learners that uses automated data mining methods to discover patterns in large data sets (Heung et al. 2016). In our case, close to 20 000 field plots on soil moisture were used to train learners to predict “wet” soils, pixel by pixel, throughout many different landscapes by combining the information in all input layers (Table 1). Figure 5 shows that the three most important variables for the random forest learner were DTW, standard deviation from elevation using a 5 cell window (which reflects local topography), and topographic wetness index from a 24 m DEM. Average summer runoff also ranked high, indicating that both very small-scale variations in local topography and large-scale variations in climate need to be considered when mapping wet areas. This agrees with other studies highlighting the complex controls of the distribution of wet soils on both local (Ågren et al. 2014) and regional scales (Jackson et al. 1999). Using ML improved performance of the wet area maps and the two best maps; random forest (Fig. 3c) and artificial neural networks (Fig. 3e) classified 84% of the “dry”/“wet” soils correctly (Table 2), with a kappa of 0.65. It should be noted that because the training dataset does not contain data from arable areas and urban areas, the models are only valid for two-thirds of the land area in Sweden, i.e. productive forest land and low-productive forest land (peatlands, pastures, thin soils and rock outcrops, areas close to and above the tree line). Here we used the Swedish productive/non-productive forests landscape as a test bench to develop a methodology of using several digital terrain indices and many thresholds together with machine learning to develop more accurate maps of wet areas. The same methodology can be used in other countries that have a high-resolution DEM and soil moisture data. Including additional terrain indices, satellite imagery and vegetation cover (Were et al. 2015; Maxwell et al. 2018) could potentially also improve the accuracy of these maps in the future.

**Table 1:** The table summarizes the GIS layers used to model the distribution of “wet” and “dry” soils with machine learners. Previous wet area maps used in forest management often consisted of just one method and threshold (DTW 1 or 2 ha stream initiation threshold has been a common approach, but other methods have also existed). By combining several terrain indices, thresholds and variability in runoff and using a training data set (NFI) that captures the distribution of “wet” soils on productive and non-productive forest lands all over the country, it is possible to generate an optimal “wet” area map across gradients in soil textures, topography and climate. This is necessary when scaling up from a catchment scale to a national scale

In-data variables layers used to classify ‘wet’ and ‘dry’ area with machine learners	Utilized scales, thresholds and periods	Source
Local topography	Moving window with 5 × 5, 10 × 10, 20 × 20, 40 × 40 and 80 × 80 grid cells	Calculated from the national 2 m DEM
Elevation above stream	Stream initiation thresholds of 0.5 ha, 1 ha, 2 ha, 5 ha, 10 ha, 15 ha and 30 ha	Calculated from the national 2 m DEM
Depth to water	Stream initiation thresholds of 0.5 ha, 1 ha, 2 ha, 5 ha, 10 ha, 15 ha and 30 ha	Calculated from the national 2 m DEM
Topographic wetness index	Resampled to a 24 m DEM and a 48 m DEM	Calculated from 24 and 48 m DEM
Quaternary deposits		From Swedish Geological Survey
Wetlands from the 1:12 500 scale property map		From Swedish Mapping, Cadastral and Land Registration Authority
Runoff	Spring, summer, autumn, winter and annual average runoff	Calculated with S-HYPE (Arheimer et al. 2011)

**Table 2:** Summary of accuracy of currently available maps and performance of the ML models when predicting the test dataset. Overall accuracy is the percentage of field plots that were correctly classified. Accuracy “wet” is the percentage of all “wet” field plots that were correctly classified as “wet” and accuracy “dry” is the percentage of all “dry” field plots that were correctly classified as “dry”. The kappa value represents the level of agreement of two dataset corrected by chance

Wet area map	Overall accuracy (%)	Accuracy “wet” (%)	Accuracy “dry” (%)	Kappa value
Wetlands from property map	74	36	99	0.39
SFA DTW map	79	73	82	0.55
ML Random forest	84	75	89	0.65
ML Support vector machine	82	68	90	0.60
ML Artificial neural network	84	74	90	0.65
ML Naïve Bayes	80	66	89	0.57

The developed maps have a high applicability and can be used to plan forest management in a way that reduces the effects on surface waters (Ågren et al. 2014). In Sweden, where cut-to-length forestry is the norm, forest soil trafficking is conducted by a harvester that cut trees to length and a forwarder that extracts timber, but also during thinning, fertilization, site preparation and harvest of logging residues for energy production (Ågren et al. 2015). This is also where the probability maps (Fig. 4a shows one of the maps with the best performance) can be used to plan off-road driving, especially the placement of extraction roads which suffer repeated heavy loads (a large laden forwarder can weigh 40 metric tons) during clear-cut. These extraction roads should not be placed in the red areas of Fig. 4a to avoid soil damage. Yellow areas in Fig. 4a are where the map is most likely to be inaccurate and extra care should be taken by the user, while green areas are more suitable for driving.

The maps can also be used to balance the green energy targets (Renewable Energy Directive) and surface water protection (EU Water Framework Directive) by planning extraction of logging residues for energy production. On “wet” soils, we recommend leaving the logging residues to reinforce the soils, by building slash mats to decrease the loads of the heavy machinery (Cambi et al. 2015) and thereby reduce the negative effects on surface waters. In “dry” areas, where soils have a higher bearing capacity, we suggest that the logging residues are harvested for bioenergy. The maps can be used in a first step of site planning but should be field validated on site during operations. There is also significant temporal variability in distributions of wet soils (Fig. 2b, c) that are not taken into account in these maps (Fig. 3). During winter when soils are frozen or during very dry conditions, it will be possible to traffic parts of the area marked as “wet”. This is something practitioners are well aware of and utilize. However, the planning can be simplified by maps that indicate the trafficability during more problematic periods where soils are wetter after snowmelt and rains. During extremely wet conditions, almost all soils become wet or moist and are more susceptible to rut formation (Mohtashami et al. 2017). Therefore, it is common to find ruts outside the areas marked as “wet” in the maps (Fig. 3b) (Ågren et al. 2015; Mohtashami et al. 2017). However, forestry operations in the “dry” areas on the map (Fig. 3) pose a smaller risk for increased sediment transport and nutrient/mercury leaching than operations in the “wet” areas where the connectivity to surface waters is higher (Ågren et al. 2015). The maps can also be used to plan hydrologically adapted protection zones near streams. Hydrologically adapted protection zones are better than using a fixed-width approach and offers an optimized site-specific riparian buffer when it comes to protection of ecological values (Gregory et al. 1991) of riparian zones (Kuglerová et al. 2014). Hydrologically adapted riparian protection zones have also been found to be more cost-effective than fixed-width zones (Tiwari et al. 2016). Hence, implementing the maps developed in this study (Figs. 3, 4) is a strategic option to meet both protection and production goals. Future research entails investigating if the maps can be used to further improve forest growth models used on a stand level or for national estimates, and whether they can be used in, for example, biogeochemical or ecological research.

## Conclusions

Here we demonstrated that machine learning can be used to create new and more accurate high-resolution maps of wet soils. These maps are better than previously used fixed threshold DTW maps. The new maps can, for example, be used to suggest machine free zones near streams and lakes in order to prevent rutting from forestry machines to reduce sediment, mercury and nutrient loads to downstream streams, lakes and sea. Further research should explore other remote sensing data such as satellite imagery or LiDAR intensity.
